# Plasticity in Standard and Maximum Aerobic Metabolic Rates in Two Populations of an Estuarine Dependent Teleost, Spotted Seatrout (*Cynoscion nebulosus*)

**DOI:** 10.3390/biology8020046

**Published:** 2019-06-14

**Authors:** Jingwei Song, Richard W. Brill, Jan R. McDowell

**Affiliations:** Virginia Institute of Marine Science, William & Mary, P.O. Box 1346, Gloucester Point, VA 23062, USA; rbrill@vims.edu (R.W.B.); mcdowell@vims.edu (J.R.M.)

**Keywords:** metabolic rates, aerobic scope, estuarine fish, plasticity

## Abstract

We studied the effects of metabolic cold adaptation (MCA) in two populations of a eurythermal species, spotted seatrout (*Cynoscion nebulosus*) along the U.S. East Coast. Fish were captured from their natural environment and acclimated at control temperatures 15 °C or 20 °C. Their oxygen consumption rates, a proxy for metabolic rates, were measured using intermittent flow respirometry during acute temperature decrease or increase (2.5 °C per hour). Mass-specific standard metabolic rates (SMR) were higher in fish from the northern population across an ecologically relevant temperature gradient (5 °C to 30 °C). SMR were up to 37% higher in the northern population at 25 °C and maximum metabolic rates (MMR) were up to 20% higher at 20 °C. We found evidence of active metabolic compensation in the southern population from 5 °C to 15 °C (Q_10_ < 2), but not in the northern population. Taken together, our results indicate differences in metabolic plasticity between the northern and southern populations of spotted seatrout and provide a mechanistic basis for predicting population-specific responses to climate change.

## 1. Introduction

Temperature has long been known to have a profound influence on the physiology and metabolic rates of fishes [1,2]. It dictates the rate of biochemical reactions at the cellular level and leads to increased metabolic rates in warm water [3,4]. The metabolic cold adaptation hypothesis (MCA) was first proposed over 100 years ago and has since been a controversial topic in fish physiology [5,6,7,8]. MCA predicts that species from colder environments (higher latitudes or altitudes) will have elevated standard metabolic rates (SMR, minimum metabolic rates needed to sustain life) compared to those from warmer climates. A meta-analysis approach found support for MCA in that fishes with ranges extending to higher latitudes have higher SMR, higher rates of mitochondrial respiration, and higher enzyme activity than counterparts living at lower latitudes [9]. Other studies, including those by Holeton (1974) and Steffensen (2002), argued that MCA is an experimental artifact and there was no evidence of elevated SMR in Arctic or Antarctic fishes when comparisons were made to similar species from temperate regions [7,8]. Within the *Fundulus notatus* species group (*F. notatus*, *F. olivaceus*, and *F. euryzonus*), evidence of MCA was found at the intraspecific level, but not at the interspecific level [10].

Climate change is predicted to bring disproportionately large impacts to coastal estuaries due to their shallow depths and proximity to human activities [11]. The metabolic physiology of eurythermal species living in these environments is much less studied compared to other economically important fishes such as cod and salmon. Anthropogenic climate change is causing increasing average water temperatures and larger temperature variation, which warrants a better understanding of the metabolic capacities and limits of estuarine fishes [12]. In addition, most studies of metabolic adaptation of fishes have treated species as a single homogeneous unit and ignored intraspecific variation in phenotypic plasticity [13,14,15]. This hinders our ability to predict a population-specific response to acute thermal change [16,17,18].

**Figure 1:**
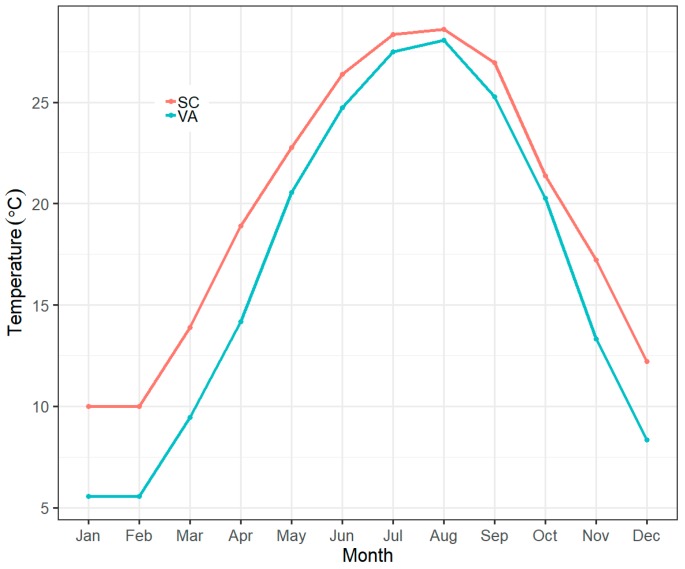
Monthly mean water temperature at two sampling locations. VA = Corrotoman River, Virginia, SC = Charleston, South Carolina.

Spotted seatrout, *Cynoscion nebulosus* (Cuvier), is a coastal species distributed from the Chesapeake Bay to the Gulf of Mexico [19]. With such a wide coastal distribution, it is possible that spotted seatrout populations inhabiting heterogeneous thermal environments have developed metabolic plasticity. At the northern range limit, spotted seatrout encounter comparatively low winter water temperatures, while the maximum water temperatures in summer are like those encountered in more southern regions (Figure 1). Periodic winter mortalities are most severe at high latitudes for these fishes and act as a strong selective pressure on cold tolerance [20]. Tagging studies have shown that dispersal distances of spotted seatrout are generally less than 50 km in the Gulf of Mexico and the southeastern coast of the U.S. [21,22,23,24,25]. Near the northern latitudinal limit of spotted seatrout in Virginia and North Carolina (hereafter the “northern population”), at least 25% of tagged fish were found to migrate over 100 km, presumably in response to changing water temperature [26]. Studies have also found differences in life history characteristics as compared to fish sampled farther south. Spotted seatrout sampled from Chesapeake Bay grow faster and mature at a larger size than their counterparts from South Carolina and Florida [27,28,29]. In addition, genetic studies indicate that the northern population is genetically distinct from spotted seatrout from regions south of the New River, North Carolina on the U.S. Atlantic coast (hereafter the “southern population”) [30,31,32]. This north–south differentiation provides an opportunity for intraspecific comparison of metabolic plasticity within a widely-distributed estuarine species.

A better understanding in the mechanistic basis of the observed differences between the two spotted seatrout populations is critical for predicting future response under climate change. We were interested in whether plasticity in metabolic phenotypes has arisen in populations of spotted seatrout, and whether the pattern is consistent with MCA. Our null hypotheses were:

1. There are no significant differences in metabolic phenotypes across a range of ecologically-relevant temperatures between the two populations as measured by SMR, MMR, factorial aerobic scope (FAS) or absolute aerobic scope (AAS) (defined as MMR/SMR and MMR-SMR, respectively);
2. There is no significant difference in thermal sensitivity of SMR (quantified as Q_10_ values) between the two populations across a range of ecologically relevant temperatures.

## 2. Materials and Methods

### 2.1. Animal Collection and Husbandry

All animal care and use protocols were approved by The College of William and Mary’s Institutional Animal Care and Use Committee (IACUC-2017-09-25-12356-jrmcdo). Spotted seatrout (*Cynoscion nebulosus*) were captured by hook and line from two locations approximately 800 km apart: (1) Corrotoman River, Virginia (*n* = 20, VA, latitude 37.732985, longitude −76.408968) and (2) areas near Charleston, South Carolina (*n* = 11, SC, latitude 32.753055, longitude −79.896670, Table 1). Both populations were sampled in November 2017, however, some SC fish died after transportation from SC to VA. The Southern population (SC) was sampled again (*n* = 6) in April 2017. Sampling dates for each fish in each site, weight, time spent in acclimation, and the test in which it was used are included in Table S1. Fish from each sampling location were acclimated in separate flow-through 10,000 L circular aquaria. Water temperature was maintained at 15 ± 1 °C and 20 ± 1 °C using heat exchangers (subsequently referred to as cold-stress experiments and heat-stress experiments, respectively). Salinity from the York River ranged from 15–22 ppt over the acclimation periods. Water quality including pH, ammonia, nitrate, and nitrite levels were checked daily using a commercial kit (Master Test Kits, API, Chalfont, PA, USA). The cold-stress experiments were conducted between January and May 2018 and the heat-stress experiments were conducted between July and August 2018. All fish were fed frozen and thawed bay anchovies (*Anchoa mitchilli*) every 2 days to satiation. Prior to an experiment, food was withheld for 48 h to ensure complete gastric evacuation [33].

### 2.2. Experimental Procedures

We used automated intermittent-flow respirometry to determine oxygen consumption rates (MO_2_, mg O_2_ kg^−1^ h^−1^) as described elsewhere [34,35]. This procedure is considered the best practice for measuring MO_2_ in fishes as it records MO_2_ with high temporal resolution, without the constant presence of a researcher [36,37].

At the start of each trial, fish were gently netted from the holding tanks and placed into either a 4 L or 7 L cylindrical respirometer (Loligo System, Viborg, Denmark) depending on the total length of the fish. The partial pressure of oxygen (PO_2_, mm Hg) in the respirometers was continuously measured with a fiber-optic oxygen meter (model FSO2-4, PyroScience, Aachen, Germany). The sensors were calibrated using two-point methods according to the manufacturer’s handbook prior to experiments and mounted in the recirculation loop of the respirometer. Each ṀO_2_ measurement was executed using a 180 s flush, 60 s wait and 180–1200 s measurement period (5 °C, 1200 s; 10 °C, 600 s; 15 °C, 300 s; 20 °C, 240 s; 25 °C, 240 s; 30 °C, 180 s). This is because fish metabolic rate correlates positively with water temperature. Longer measurement periods were needed at lower temperatures for fish to consume a similar amount of oxygen at higher temperatures. The operation of the system and data recording was done via the AquaResp software (available at: www.aquaresp.com).

Individual fish in the cold-stress group were exposed to three water temperatures: 15 °C (20 h), 10 °C (5 h) and 5 °C (18 h) in each experiment. Experiments on individuals from the heat-stress group were also exposed to three water temperatures: 20 °C (20 h), 25 °C (5 h) and 30 °C (18 h). The acute decreases or increases between temperature steps were completed within 2 h. The range of temperatures is representative of the temperature limits of the estuarine environment spotted seatrout occupy. Because of the limited availability of specimens from SC, the last six fish were returned to the holding tank after the cold-stress experiment and re-acclimated for at least 30 days at 20 °C before the heat-stress experiments (all fish survived the acute temperature changes, except for a single fish from SC, which died during a heat stress experiment due to equipment failure). Between trials, respirometers and connecting tubing were thoroughly cleaned with bleach and rinsed with a large amount of fresh water. Bacterial background respiration was measured after fish were removed at the end of cold-stress or heat-stress experiments (5 °C, 30 °C, respectively). Background respiration values were negligible (< 1% of the rate of oxygen decline recorded when fish were present) during both the cold-stress and heat-stress experiments and were subsequently ignored.

MO_2_ for a given measurement period was calculated from the time course of oxygen partial pressure (PO_2_) change: MO_2_ = VΔPO_2_Δt^−1^β, where V is the respirometer volume (L) corrected for fish volume (assuming 1 kg = 1 L), ΔPO_2_Δt^−1^ is the slope of the linear regression (mm Hg·h^−1^), and β is the oxygen solubility coefficient (mg O_2_ mm Hg^−1^·L^−1^) [38]. Slopes with r^2^ < 0.95 were excluded from analyses.

### 2.3. Correcting for Body Weight

To compare the metabolic rates between different fish, all values were normalized to a standard body weight (the mean body weight of all fish used in the experiments, which equaled 0.34 kg). The following formula was used:

$$MO_{2,std}=MO_{2,obs}{\left(\frac{\mathrm{BW}}{0.34}\right)}^{\left(1-b\right)}$$

where $MO_{2,std}$ is the mass-specific oxygen consumption rate (mg O_2_ kg^−1^ h^−1^), $MO_{2,obs}$ is the observed oxygen consumption rate (mg O_2_ kg^−1^ h^−1^), and BW is the actual weight (kg) of the fish. The range of body weight of experimental animals was too small (less than an order of magnitude) to determine the mass exponent *b* [39]. Therefore, we used *b* = 0.948, which is the average value using all available data on teleost to correct for SMR [40]. MMR was corrected using the same formula but with *b* = 0.937 [40].

### 2.4. Data Analysis and Statistical Procedures

**Figure 2:**
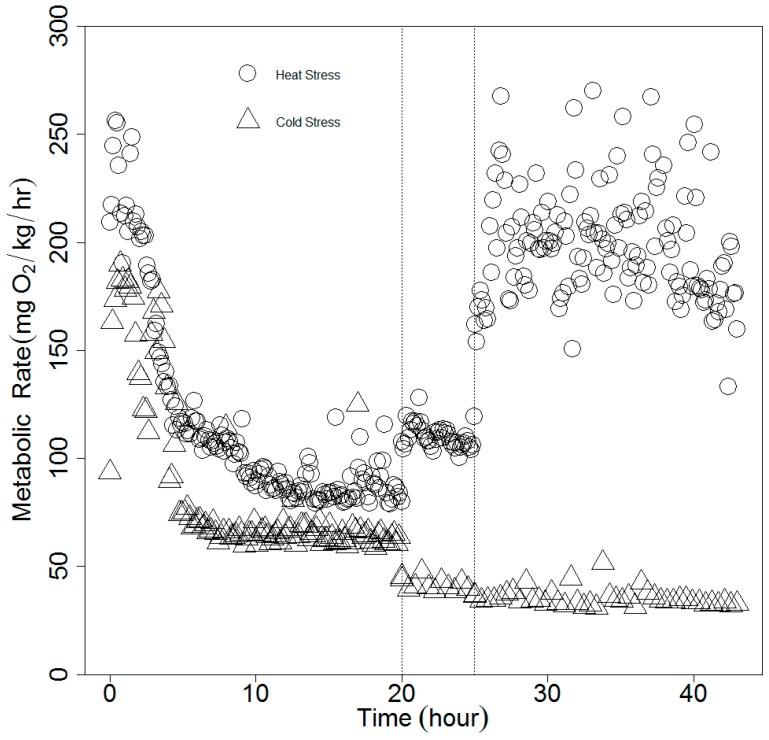
Time course of metabolic rate change in spotted seatrout under two sets of experimental conditions. Empty circles: heat stress; empty triangles: cold stress. Each dot represents the oxygen consumption rate from one respirometry cycle. Vertical dotted lines separate different temperatures. Within each treatment, from left to right: 20 °C, 25 °C, 30 °C (heat stress); 15 °C, 10 °C, 5 °C (cold stress).

We defined SMR as the minimum metabolic rate of a post-absorptive fish at a given temperature. At the starting temperatures, 15 °C and 20 °C, MO_2_ was initially elevated because of handling stress and gradually declined to SMR (Figure 2). We used the mclust package [41] implemented in R [42], which fits a mixture of normal distributions to the data [41]. For each fish, the mean of the lowest normal distribution was taken as the SMR [43].

MMR was defined as the single highest MO_2_ value at 15 °C and 20 °C for individual fish. We calculated two measures of aerobic scope: absolute aerobic scope (AAS) and factorial aerobic scope (FAS). The former is the difference between MMR and SMR, and the latter is calculated as the ratio of MMR/SMR [44]. We quantified thermal sensitivity by Q_10_ values (i.e., the factor by which the rate of a biochemical process changes over a 10 °C change in temperature) using the following formula [45]:

$$Q_{10}={\left(\frac{R_{2}}{R_{1}}\right)}^{\left(\frac{10}{t_{2}-t_{1}}\right)}$$

where R_1_ and R_2_ are the SMR at temperature t_1_ and t_2_ (t_1_ + 10 °C), respectively.

We used the nlme package [46] implemented in R [42] to perform a linear mixed effects analysis of the relationship between SMR and origin of the fish. As fixed effects, we entered fish origin, temperature and their interaction term into the model. We used individual fish as a random effect. *p*-values were obtained by likelihood ratio tests of the full model with the effect in question against the model without the effect in question. Two-tailed Student’s *t*-test was used to compare means between groups. A significance level of *α* = 0.05 was used for all statistical tests.

## 3. Results

### 3.1. SMR

**Figure 3:**
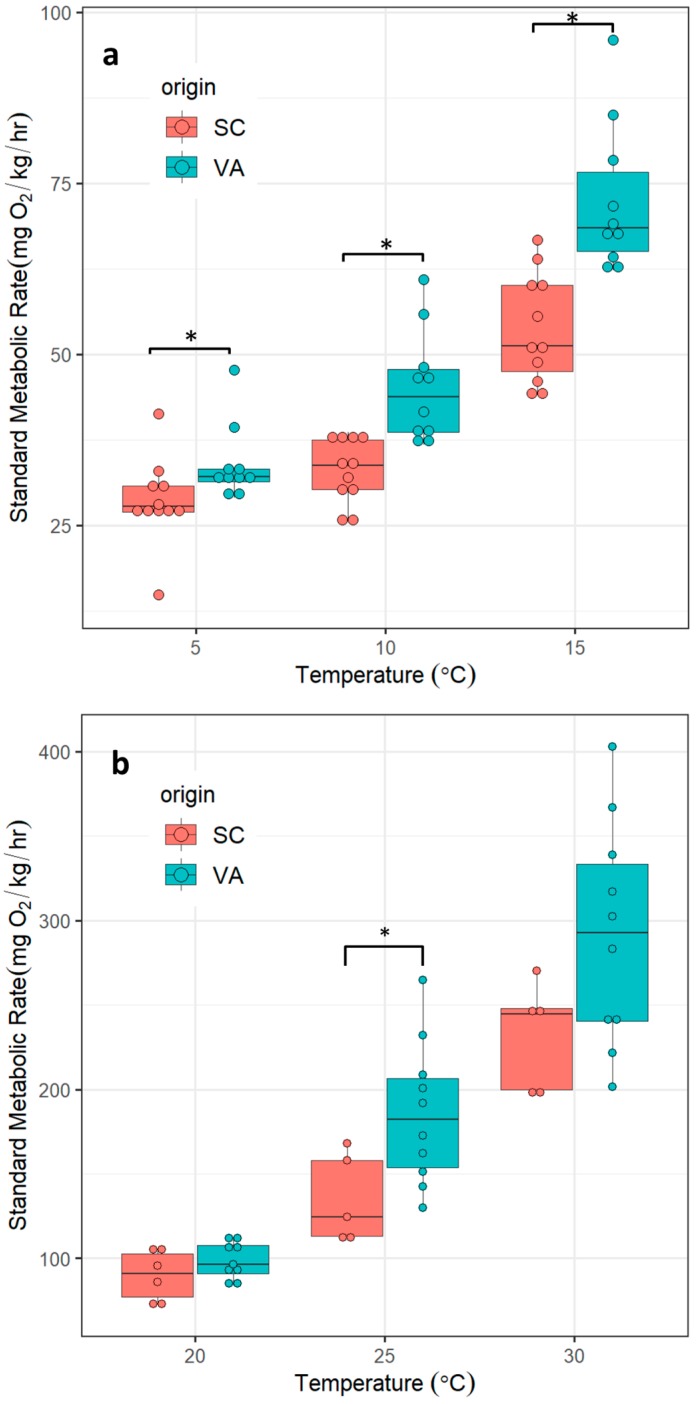
SMR of spotted seatrout from two genetically distinct populations measured at six discrete temperatures. SC = southern population, VA = northern population. (**a**) cold stress; (**b**) heat stress. Asterisks indicate significant differences between groups (*p* ≤ 0.05, two tailed Student’s t-test).

SMR in both VA and SC spotted seatrout increased exponentially from 5 °C to 30 °C. SMR in SC ranged from 24 to 233 mg O_2_ kg^−1^·h^−1^; VA ranged from 28 to 349 mg O_2_ kg^−1^·h^−1^. Mean SMR between the northern and southern populations differed significantly at 5 °C (t_19_ = 2.1), 10 °C (t_19_ = 4.25), 15 °C (t_19_ = 4.53), 25 °C (t_13_ = 2.42) but not at 20 °C (t_13_ = 1.41) or 30 °C (t_13_ = 1.90) (Figure 3). Developmental plasticity and seasonality could cause SMR to differ between batches of fishes sampled at different times. To test if this effect was detectable in SC fish sampled at different time periods, SMR was plotted separately for SC fish sampled from the second time period. No significant differences were detected (Figure S1), therefore data from two groups of SC were pooled in subsequent analyses.

In cold stress experiments, the origin of fish had a significant effect on SMR (χ^2^ (1) = 8.97, *p* = 0.0027), as did temperature (χ^2^ (2) = 104.06, *p* < 0.0001). The fish–temperature interaction was also significant (χ^2^ (2) = 20.14, *p* < 0.001). Contrasts revealed that: (1) the effect of temperature on SMR was significantly larger in individuals from the northern population versus individuals from the southern population (5 °C to 10 °C: *b* = −6.65, *t_38_* = −4.121, *p* < 0.001; 10 °C to 15 °C: *b* = −6.59, *t*_38_ = −4.17, *p* < 0.001).

In heat stress experiments, the origin of fish had no significant effect on SMR (χ^2^ (1) = 2.48, *p* = 0.12), perhaps due to a small sample size in the SC group. Temperature had a significant effect on SMR (χ^2^ (2) = 69, *p* < 0.0001). Origin–temperature interaction did not significantly improve the fit of the model (χ^2^ (2) = 3.54, *p* = 0.17). Predicted SMR in spotted seatrout originating from the northern population was up to 37% higher than SMR in spotted seatrout from the southern population at 25 °C (185.6 vs. 135.1 mg O_2_ kg^−1^·h^−1^, respectively). The smallest difference was at 5 °C, with fish from the northern population showing an approximately 19% higher SMR than fish from the southern population (34 vs. 28.6 mg O_2_ kg^−1^·h^−1^, respectively) (Table 2).

### 3.2. Maximum Metabolic Rates

**Figure 4:**
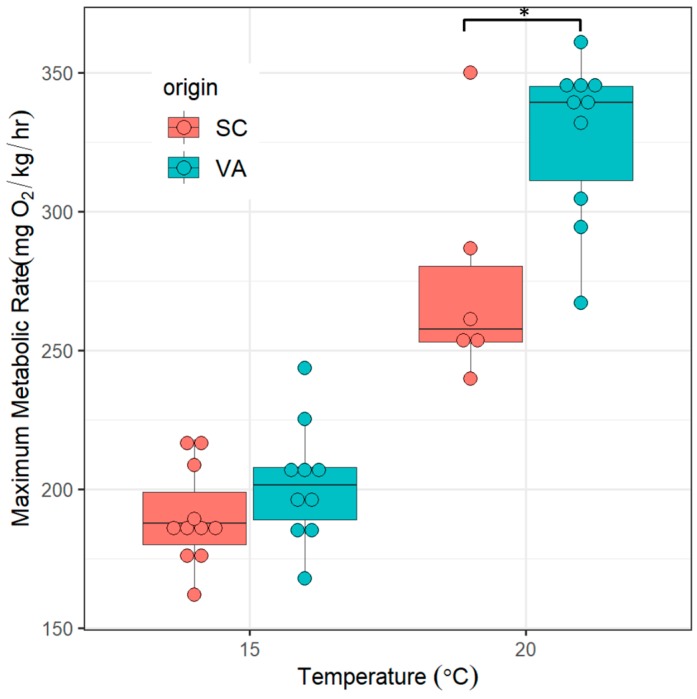
MMR of spotted seatrout from two genetically distinct populations measured at 15 °C and 20 °C. SC = southern population, VA = northern population. Asterisks indicate significant differences between groups (*p* ≤ 0.05, two tailed Student’s t-test).

Mean MMR between northern and southern spotted seatrout was not significantly different at 15 °C (t_19_ = 1.38, *p* = 0.18), but was significantly different at 20 °C (t_14_ = 3.07, *p* = 0.008) (Figure 4).

### 3.3. Aerobic Scope

**Figure 5:**
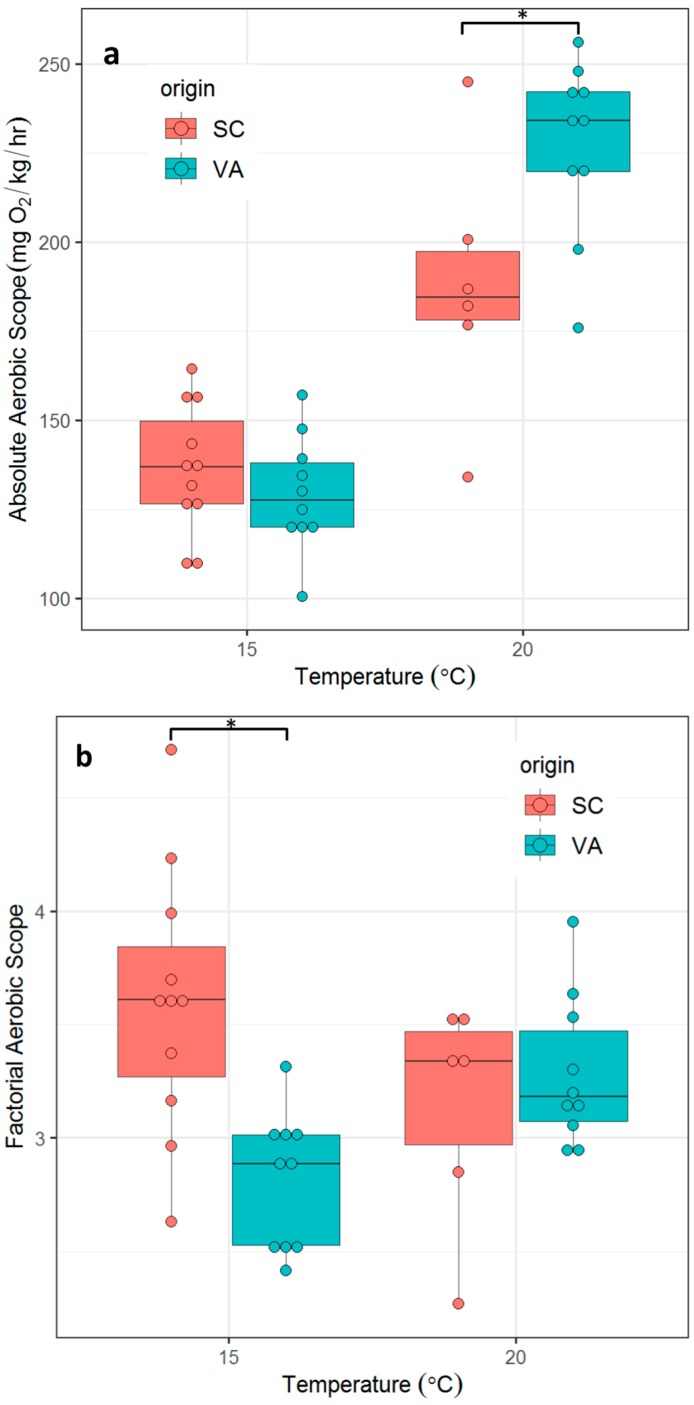
Absolute aerobic scope (AAS, **a**) and factorial aerobic scope (FAS, **b**) between two northern and southern spotted seatrout populations, at 15 °C and 20 °C. SC = southern population, VA = northern population. Asterisks indicate significant differences between groups (*p* ≤ 0.05, two tailed Student’s t-test).

Mean AAS of fish from the northern population was significantly higher at 20 °C (t_14_ = 2.62, *p* = 0.01) but not at 15 °C (t_19_ = 0.93, *p* = 0.366). For FAS, the differences were reversed. Mean FAS of fish from the southern population was significantly higher at 15 °C (t_19_ = 3.93, *p* < 0.001) than mean FAS of fish from the northern population, but not at 20 °C (t_14_ = 0.87, *p* = 0.4) (Figure 5).

### 3.4. Q_10_

**Figure 6:**
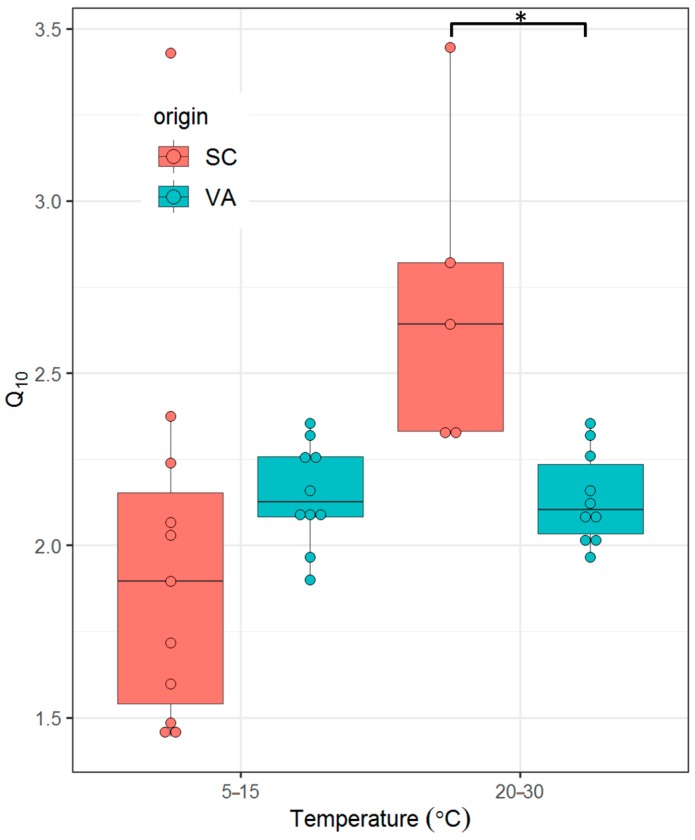
Temperature coefficient (Q_10_) of spotted seatrout between northern and southern spotted seatrout populations subjected to cold stress (5 to 15 °C) and heat stress (20 to 30 °C). SC = southern population, VA = northern population. Asterisks indicate significant differences between groups (*p* ≤ 0.05, two tailed Student’s t-test).

The Q_10_ ranged between 1.4 and 3.4 among SC fish and 1.9 to 2.4 among VA fish. There were no significant differences in Q_10_ between the two populations at temperatures between 5 °C and 15 °C, (t_18_ = 1.2, *p* = 0.32). There was, however, a significant difference in Q_10_ between the two populations between 20 °C and 30 °C (t_13_ = 3.77, *p* = 0.0025) (Figure 6).

## 4. Discussion

Estuaries are characterized by large daily and seasonal temperature fluctuations [47] and we argue that spotted seatrout have evolved metabolic plasticity to cope with the challenge of living in these dynamic environments. The degree of physiological plasticity can, however, also vary among populations inhabiting heterogeneous environments [48,49]. Using cold- and heat-stress experiments, we showed that spotted seatrout can maintain a broad range of metabolic rates from 5 °C to 30 °C. In addition, we found that the reaction norms of metabolic responses to acute temperature changes are population-specific, with northern fish having higher SMR than southern fish, consistent with the predictions of MCA [50]. Similar patterns have been observed in another estuarine-dependent species, the common killifish (*Fundulus heteroclitus*); fish sampled from a northern population (New Hampshire, USA) were found to have a higher metabolic rate than fish sampled from a southern population (Georgia, USA) [51]. Our sampling locations were in closer proximity to each other as compared to the killifish study (800 km vs. 1800 km). Thus, the observed differences in SMR between spotted seatrout sampled from different thermal regimes provide evidence that intraspecific metabolic plasticity can take place at a spatial scale of a few hundred kilometers and suggest that temperature may play an important role in the maintenance of genetic breaks. It should be noted that our sample size in the SC heat stress group was relatively low (*n* = 5) due to difficulty in obtaining live specimens. This may have had an effect on our ability to detect statistical significance in SMR between SC and VA at 20 and 30 °C (Figure 3). Nevertheless, there were significant differences between SC and VA in the heat stress group based on MMR, AAS and Q_10_ despite a small sample size, consistent with differences in metabolic phenotypes between northern and southern spotted seatrout.

SMR appear to approach a threshold at the lowest experimental temperature, 5 °C, in both spotted seatrout populations and this threshold likely limits overwinter survival of this species at its northern distributional limit. This finding is consistent with the results of both telemetry studies and previous cold tolerance experiments. In North Carolina, all winter mortality events of telemetered spotted seatrout in their natural environment occurred in water temperatures below 7 °C, and a precipitous increase in natural mortality occurred at water temperatures below ~ 4 °C [52]. Cold tolerance experiments using fish from both North Carolina and South Carolina show that when fish are exposed to water temperatures below ~4 °C, survival is short-term and physiological impairments due to acute cold stress are largely irreversible [53,54].

Organisms with a larger AS at a given temperature are considered to be more capable of performing energetically demanding tasks such as growth [55,56,57]. Previous studies found that juvenile spotted seatrout in Chesapeake Bay reach larger sizes at maturity than fish from South Carolina, despite a growing season in Chesapeake Bay that is about a month shorter [27,29,58]. We hypothesized that a larger AS of VA spotted seatrout might compensate for a shorter growing season. AS is most commonly represented as AAS in fishes, but FAS is also used (albeit less frequently) [44,59,60]. AAS provides an exact value for metabolic rates above SMR while FAS accounts for the fact that different individuals require proportionally different rates of oxygen delivery to the tissues to perform a given physiological task [44]. Here we report both measures of AS, but base our interpretation on AAS. Our data show that the standard deviation in percentage of the mean value is lower for MMR than SMR and AAS has been found to be more robust when the variability in SMR is larger than that of MMR [44] (Table S2). Our experiments obtained MMR at 15 °C and 20 °C and therefore AAS at these two temperatures. Both temperatures are in the middle of the wide range of temperatures that spotted seatrout tolerates and representative of temperatures in their natural habitats [61]. AAS was significantly larger in VA than SC fish at 20 °C. Thus, the faster growth of juvenile spotted seatrout in Chesapeake Bay is likely achieved when water temperatures are ~ 20 °C. It is unclear if AAS is also larger in temperatures over 20 °C or between 15 °C and 20 °C.

The temperature sensitivity (i.e., Q_10_) of metabolic rates differs among species and even populations and reflects physiological adaptations [62]. Q_10_ values in this study ranged from 1.4 to 3.4. This is in general agreement with values commonly reported in teleost fishes across a broad range of temperatures (2–3) [63]. The finding that some southern spotted seatrout show mean Q_10_ < 2 at 5–15 °C suggests active metabolic compensation, while there is no evidence of such compensation in the northern population (mean Q_10_ > 2). A previous study comparing seasonal metabolic rates of spotted seatrout with its congener sand seatrout (*Cynoscion arenarius*) found that the former shows a greater degree of metabolic compensation (i.e., lower Q_10_ values) from 15 °C to 30 °C [64]. Spotted seatrout are year-round residents in estuaries, whereas sand seatrout migrate offshore in the winter months [65]. The authors concluded that the lower Q_10_ value observed in spotted seatrout allow them to remain active in the estuarine environment during the warmest months in Texas estuaries [64]. Analogous to the congenic comparison, Q_10_ is significantly lower in VA than SC spotted seatrout between 20 °C and 30 °C. This may allow VA fish to survive similar maximum water temperature in the summer, despite the fact that higher SMR might be detrimental at higher temperatures.

Whether the observed differences in metabolic phenotypes have a genetic basis in spotted seatrout is unknown. Future studies should use a common garden experimental design in which metabolic rates are measured over multiple generations to rule out effects from ontogeny or an individual’s thermal history [66,67]. Although spotted seatrout can reach sexual maturity relatively quickly compared to other sciaenid fishes (age 1), such experiments would still require multiple years to complete. Alternatively, genome scan approaches can be used to look for functional loci that show unusually large differentiation between populations, which can serve as indirect evidence for the genetic basis of whole-organism physiological differences [68,69]. For example, a study combined physiological tests and genomic analyses to examine thermal adaptation between conspecific populations of redband trout [70]. Populations from a desert climate (hot) showed improved cardiorespiratory capacity at high test temperatures compared to a population from a montane climate (cool). In addition, genomic and transcriptomic analyses revealed candidate genes that showed differential expressions between populations, providing additional evidence that the observed physiological differences had a heritable component.

Our results support the MCA hypothesis at the intraspecific level in spotted seatrout. Organisms with greater plasticity will likely be more resilient to an accelerated rate of environmental changes, especially temperatures [71,72]. There are increasing efforts to predict future range shifts in fishes based on physiological abilities and tolerances [73,74]. Failure to account for physiological plasticity among local populations could over- or underestimate the potential for migration and compromise the utility of these species distribution models. We contend, therefore, that our results should be explicitly incorporated into projects aiming at predicting range shifts of spotted seatrout in response to climate change.

## 5. Conclusions

The results of this study are consistent with the predictions of the MCA hypothesis. Northern populations of spotted seatrout were found to have greater metabolic plasticity than its southern counterparts when compared at ecologically relevant water temperatures. This finding can be explicitly incorporated into modeling framework to predict range shifts of spotted seatrout in response to climate change.

## Figures and Tables

**Table 1:** Number and weight of spotted seatrout used in the study. M = mean (and range) body mass in grams.

Sampling Location	Cold Stress	Heat Stress
Corrotoman River, Virginia	*n* = 10 M = 192 g (140–252 g)	*n* = 10 M = 238 g (122–439 g)
Charleston, South Carolina	*n* = 11 M = 467 g (235–838)	*n* = 5 M = 454 g (368–508)

**Table 2:** Predicted SMR from linear mixed effect model. SC = southern population, VA = northern population. Fit = predicted SMR, sd = standard deviation, se = standard error, ci = 95% confidence interval.

Origin	Temp. (°C)	N	fit	sd	se	ci
SC	5	11	28.6	5.7	1.7	3.8
SC	10	11	33.1	4.5	1.4	3.1
SC	15	11	53.8	7.1	2.1	4.8
VA	5	10	34.0	5.7	1.8	4.1
VA	10	10	45.2	7.8	2.5	5.6
VA	15	10	72.5	10.0	3.2	7.2
SC	20	5	86.6	13.4	6.0	16.6
SC	25	5	135.1	24.3	10.9	30.1
SC	30	5	231.7	29.4	13.1	36.5
VA	20	10	100.2	10.7	3.4	7.7
VA	25	10	185.6	38.3	12.1	27.4
VA	30	10	291.7	59.4	18.8	42.5

## Supplementary Materials

The following are available online at https://www.mdpi.com/2079-7737/8/2/46/s1, Figure S1: SMR of spotted seatrout sampled from SC at two different time periods: SC 1 (*n* = 5, sampled Nov 2017) and SC 2 (*n* = 6, sampled Mar 2018). Table S1: Details on the spotted seatrout specimens used in this study. Table S2: Summary of mass-specific SMR and MMR for both SC and VA spotted seatrout populations.
