# Supplementary material for: Plasticity in Standard and Maximum Aerobic Metabolic Rates in Two Populations of an Estuarine Dependent Teleost, Spotted Seatrout (Cynoscion nebulosus)
Source: Biology (Basel). 2019 Jun 14;8(2):46. doi: 10.3390/biology8020046 (PMC6627818; doi:10.3390/biology8020046)
Supplement: Supplementary file 1 [file biology-08-00046-s001.zip › Figure S1.docx]

.
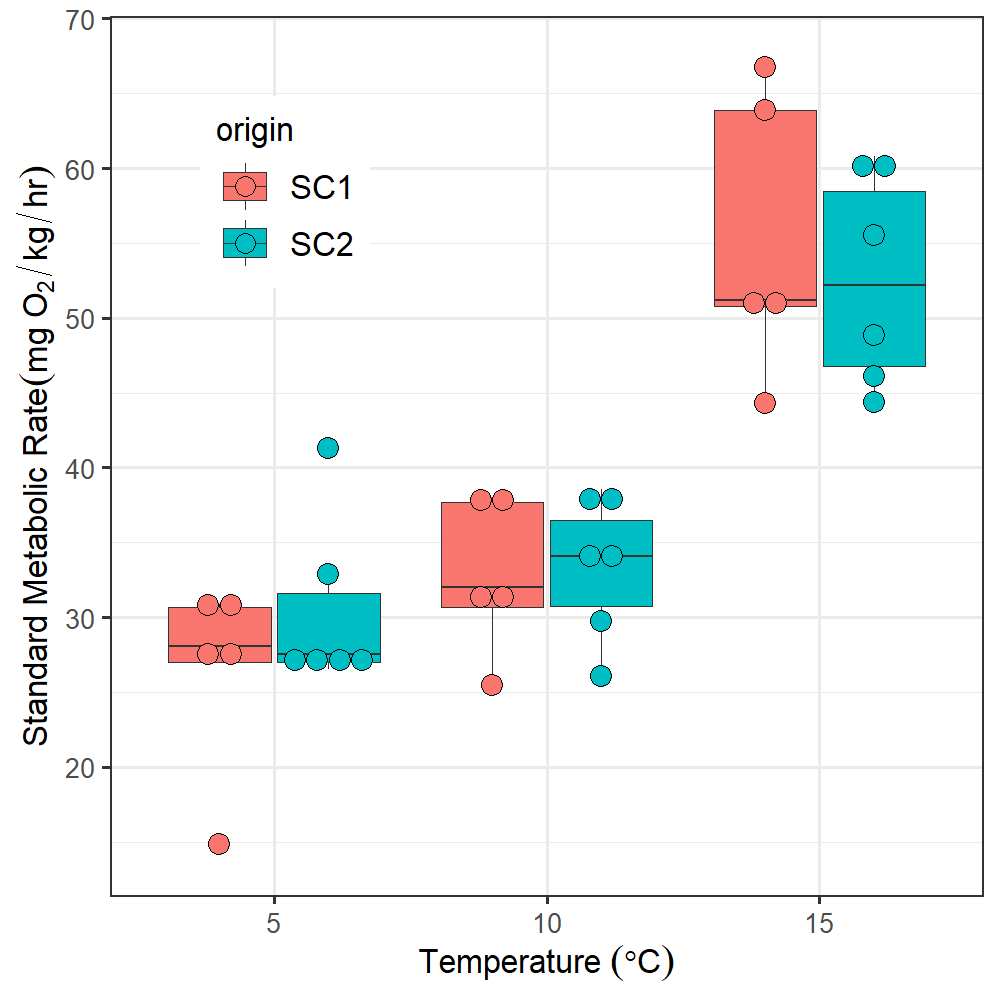


Figure S1. SMR of spotted seatrout sampled from SC at two different time periods: SC1 (n=5, sampled Nov 2017) and SC2 (n=6, sampled Mar 2018).
